# Food‘to’go - a feasibility study of post-discharge delivery of protein- and energy-enforced meals for older patients by the use of information and communications technology (ICT)

**DOI:** 10.1186/1472-6955-14-S1-S9

**Published:** 2015-10-08

**Authors:** Tove Lindhardt

**Affiliations:** 1 Copenhagen University Hospital Herlev, Denmark, and Faculty of Medicine, Lund University, Lund, Sweden

## Background

Older patients are often excluded from e-health projects on account of low IT-literacy levels. This is a barrier for their access to many current and future health care offers. The Food‘n’Go research - an innovation programme - aims to develop, test and implement information technology solutions to increase participation and empowerment of older patients and their families, in relation to frequent problems during and following hospitalisation. The present feasibility study is part of this programme.

## Aim

To test the feasibility and effectiveness of ICT-supported energy- and protein-enforced home-delivered main and in-between meals on older patients discharged from acute hospitalisation in medical wards.

## Methods

A quasi-experimental, non-randomised, controlled feasibility study. The sample consisted of older patients (n=36), 65+ years, admitted to department of internal medicine with a nutritional risk score of 3 or more, discharged to own home.

Intervention: Enriched meals were delivered to patients' homes 12 weeks after discharge and a tablet computer to order meals and register intake. Primary effect measure: Muscle strength. Secondary: BMI, dietary intake, HRQoL (EQ5D), depression (GDS5); readmissions, mortality.

## Results

Technology challenges were not related to participants, but to: the out-of hospital setting; organisational issues such as collaboration between kitchen, research sites and technology staff; and to IT-stability. Although weaker at baseline the intervention group increased their muscle strength in arms and legs more consistently than the control group at 6 and 12 weeks. They further improved their depression score, reported increased HRQoL, increased intake and appetite, and increased energy. Relatives reported positive impact on their level of worry and on the relationship between patient and relatives. NCT02268721.

